# Supplementary material for: Astrocytes enable amygdala neural representations supporting memory
Source: Nature. 2026 Feb 11;652(8109):434–41. doi: 10.1038/s41586-025-10068-0 (PMC13061616; doi:10.1038/s41586-025-10068-0)
Supplement: Supplementary file 1 — Reporting Summary [file 41586_2025_10068_MOESM1_ESM.pdf]

nature portfolio

Corresponding author(s): Andrew Holmes

Last updated by author(s): December 10, 2025

Reporting Summary

Nature Portfolio wishes to improve the reproducibility of the work that we publish. This form provides structure for consistency and transparency in reporting. For further information on Nature Portfolio policies, see our Editorial Policies and the Editorial Policy Checklist.

Statistics

For all statistical analyses, confirm that the following items are present in the figure legend, table legend, main text, or Methods section.

n/a	Confirmed	
☐	☒	The exact sample size (*n*) for each experimental group/condition, given as a discrete number and unit of measurement
☐	☒	A statement on whether measurements were taken from distinct samples or whether the same sample was measured repeatedly
☐	☒	The statistical test(s) used AND whether they are one- or two-sided<br>*Only common tests should be described solely by name; describe more complex techniques in the Methods section.*
☐	☒	A description of all covariates tested
☐	☒	A description of any assumptions or corrections, such as tests of normality and adjustment for multiple comparisons
☐	☒	A full description of the statistical parameters including central tendency (e.g. means) or other basic estimates (e.g. regression coefficient) AND variation (e.g. standard deviation) or associated estimates of uncertainty (e.g. confidence intervals)
☐	☒	For null hypothesis testing, the test statistic (e.g. *F*, *t*, *r*) with confidence intervals, effect sizes, degrees of freedom and *P* value noted<br>*Give P values as exact values whenever suitable.*
☒	☐	For Bayesian analysis, information on the choice of priors and Markov chain Monte Carlo settings
☒	☐	For hierarchical and complex designs, identification of the appropriate level for tests and full reporting of outcomes
☒	☐	Estimates of effect sizes (e.g. Cohen's *d*, Pearson's *r*), indicating how they were calculated

Our web collection on statistics for biologists contains articles on many of the points above.

Software and code

Policy information about availability of computer code

Data collection	Fiber photometry, cellular resolution, in vivo and in vitro electrophysiology data was acquired using Tucker-Davis Technologies Synapse v88 or higher, Inscopix IDAS HD v2.0.0 or higher, Plexon Omniplex1.21 and Cineplex3.5, Molecular Devises pCLAMP11.0 software. Behavioral data was acquired using Med Associates VideoFreeze2.7.0 and MedPC IV, Noldus Ethovision14 software. Images were acquired using Leica Aperio VERSA12.5, Zeiss ZEN3.10, Olympus CellSens Standard 1.15 and VS200 ASW 3.4.1, Nikon NIS-Elements 5.22.x, Keyence BZ-X Viewer software.
Data analysis	Data were analyzed using MATLAB2018a or higher, Python3.10 or higher, Inscopix IDPS v1.6, NeuroExplorer v5 , Prism8.1.0 or higher, EzTrack1.3.5, AQuA, Fiji v2.9.0 and ImageJ v1.37 software

For manuscripts utilizing custom algorithms or software that are central to the research but not yet described in published literature, software must be made available to editors and reviewers. We strongly encourage code deposition in a community repository (e.g. GitHub). See the Nature Portfolio guidelines for submitting code & software for further information.

Data

Policy information about availability of data

All manuscripts must include a data availability statement. This statement should provide the following information, where applicable:

- Accession codes, unique identifiers, or web links for publicly available datasets
- A description of any restrictions on data availability
- For clinical datasets or third party data, please ensure that the statement adheres to our policy

All source data are available on request to the corresponding author and are also available in Figshare. Custom code and program-provided code in MATLAB, Inscopix and Python software used for analysis, are available upon request from the corresponding author.

April 2023

Research involving human participants, their data, or biological material

Policy information about studies with human participants or human data. See also policy information about sex, gender (identity/presentation), and sexual orientation and race, ethnicity and racism.

Reporting on sex and gender	N/A
Reporting on race, ethnicity, or other socially relevant groupings	N/A
Population characteristics	N/A
Recruitment	N/A
Ethics oversight	*Identify tN/he organization(s) that approved the study protocol.* N/A

Note that full information on the approval of the study protocol must also be provided in the manuscript.

Field-specific reporting

Please select the one below that is the best fit for your research. If you are not sure, read the appropriate sections before making your selection.

☒ Life sciences ☐ Behavioural & social sciences ☐ Ecological, evolutionary & environmental sciences

For a reference copy of the document with all sections, see nature.com/documents/nr-reporting-summary-flat.pdf

Life sciences study design

All studies must disclose on these points even when the disclosure is negative.

Sample size	Sample size were based on pilot and previously (similar types of) experiments and were not statistically predetermined. Experiments were replicated in multiple subjects and powered to match sample sizes typical of the technique reported in the field, though no formal power analysis was performed a priori. Images shown were selected from multiple independent samples (i.e., different animals)
Data exclusions	Mice with freezing scores greater than three standard deviations above the group mean were excluded from the analysis. Mice with absent viral expression or improperly targeted implantations were excluded from analysis.
Replication	Experiments were replicated across multi-animal batches (animal N and n numbers for each experiment are provided in the corresponding figure legends and Supplementary Table 1)
Randomization	Experiments groups were randomized.
Blinding	Experimenters were blinded to group allocation whenever possible.

Reporting for specific materials, systems and methods

We require information from authors about some types of materials, experimental systems and methods used in many studies. Here, indicate whether each material, system or method listed is relevant to your study. If you are not sure if a list item applies to your research, read the appropriate section before selecting a response.

Materials & experimental systems

n/a	Involved in the study
☐	☒ Antibodies
☒	☐ Eukaryotic cell lines
☒	☐ Palaeontology and archaeology
☐	☒ Animals and other organisms
☒	☐ Clinical data
☒	☐ Dual use research of concern
☒	☐ Plants

Methods

n/a	Involved in the study
☒	☐ ChIP-seq
☒	☐ Flow cytometry
☒	☐ MRI-based neuroimaging

Antibodies

Antibodies used

Anti-GFP (chicken, Abcam catalog # ab13970), anti-TdTom (rat, Kerafast catalog # EST203), anti-DsRed (rabbit, Clontech catalog # 632496), anti-GFAP (guinea pig, Synaptic Systems catalog # 173400), Anti-S100 beta (rabbit, Abcam catalog # ab52642), anti-NeuN (rabbit, Abcam catalog # ab104225), goat anti-rabbit Alexa 405 (catalog # A31556, Life Technologies), goat anti-guinea pig Alexa 555 (catalog # A21435, Life Technologies), goat anti-rat Alexa 555, (catalog # A21434, Life Technologies), goat anti-guinea pig Alexa 647 (catalog # A21450, Life Technologies), goat anti-rabbit Alexa 680 (catalog # A21076, Life Technologies), mouse anti-GFP (catalog # A-11120, lot # 1859591, Invitrogen), anti-DsRed (rabbit, catalog # 632496, lot # 1904182; Takara Bio), biotinylated goat anti-mouse antibody (catalog # 31802, Invitrogen), goat anti-rabbit secondary antibody (catalog # A32731, Invitrogen)

Validation

Reactivity with mouse in fluorescent immunohistochemistry was confirmed by the manufacture and by several previous publication (Adamsky et al., 2018; Vaidyanathan et al., 2021; Hagihara et al., 2021)

Animals and other research organisms

Policy information about studies involving animals; ARRIVE guidelines recommended for reporting animal research, and Sex and Gender in Research

Laboratory animals

Mus musculus, males C57/Bl6J (Jakson Lab catalog #00664), Vglut1-Cre (Jakson Lab catalog #023527), and Glt1-G-CaMP7 (RIKEN BioResource Research Center catalog # G7NG817) aged 2-3 months at the time of injection (2-7 months at time of the experiment) were used.

Wild animals

This study did not involve wild animals

Reporting on sex

Males animals were used in this study.

Field-collected samples

This study did not involve samples collected from the field.

Ethics oversight

Experimental procedures were approved by the NIAAA and National Institute of Natural Sciences Animal Care and Use Committees and followed the NIH guidelines outlined in ‘Using Animals in Intramural Research’ and the local Animal Care and Use Committees.

Note that full information on the approval of the study protocol must also be provided in the manuscript.

Plants

Seed stocks

N/A

Novel plant genotypes

N/A

Authentication

N/A
