# Supplementary material for: Astrocytes enable amygdala neural representations supporting memory
Source: Nature. 2026 Feb 11;652(8109):434–41. doi: 10.1038/s41586-025-10068-0 (PMC13061616; doi:10.1038/s41586-025-10068-0)
Supplement: Supplementary file 2 — Summary table of statistical tests and results. [file 41586_2025_10068_MOESM2_ESM.docx]

**Table S1**: Statistical results. Experiments listed in order of appearance in the main text of the manuscript. Abbreviations (in alphabetical order): ANOVA; analysis of variance, AQuA; astrocyte quantification and analysis, AUC; area under the curve, BLA; basolateral amygdala, bCI; bootstrap confidence intervals, Ca^2+^; calcium, CalEx, calcium extruder plasma membrane calcium ATPase, Clozapine-n-oxide; CNO, CS; conditioned stimulus, cyto-GCaMP6f; GCaMP6f expressed in astrocyte cytosol, Ext; extinction, E-Ext; early extinction, L-Ext; late extinction, E-Ret; extinction retrieval, F-Con; fear conditioning, F-Ren; fear renewal, F-Ret; fear retrieval, GFAP; glial fibrillary acidic protein, hSyn-GCaMP7f; GCaMP7f expressed in neurons, hM4Di; modified form of the human M4 muscarinic receptor, hM3Dq; modified form of the human M3 muscarinic receptor; lck-GCaMP6f; GCaMP6f expressed in astrocyte processes, NeuN; neuronal nuclear antigen, sEPSC; spontaneous excitatory postsynaptic current, PL; prelimbic prefrontal cortex, PT; permutation test, US; unconditioned stimulus, Veh; vehicle. *P* values are shown to a maximum value of *P*=0.0001. Significant results highlighted by *

| Related to **Fig. 1** | | |  |
| --- | --- | --- | --- |
| **Fig. 1b** | | |  |
|  | *%GFP+ cells* | |  |
| Cyto-GCaMP6f expressing cell counts (n=16 sections/6 mice) | GFAP+ vs NeuN+ cells  2-tailed paired *t*-test: *t*(15) = 67.65, *P* < 0.0001* | |  |
|  | | |  |
| **Fig. 1d** | | |  |
|  | *% Freezing* | |  |
| Fiber photometry (n=17 mice) | Repeated measures 1-way ANOVA *F*(4.462, 71.39) = 29.89, *P* < 0.0001*  Šídák’s *post hoc* tests:  F-Con CS3 vs CS1 *P* = 0.0001*; E-Ext vs L-Ext *P* < 0.0001*; E-Ext vs E-Ret *P* < 0.0001*; F-Con CS3 vs F-Ren *P* = 0.6022 | |  |
|  | | |  |
| **Fig. 1e** | | |  |
|  | *Peri-event Ca^2+^ activity during fear conditioning* | |  |
| Fiber photometry (n=17 mice) | bCI denoted as green lines | |  |
|  | | |  |
| **Fig. 1f** | | |  |
|  | *AUC of peri-event Ca^2+^ activity during fear conditioning* | |  |
| Fiber photometry (n=17 mice) | F-Con CS vs US, 2-tailed paired *t*-test: t(16) = 3.027, *P* = 0.0080* | |  |
|  | | |  |
| **Fig. 1g** | | |  |
|  | *Peri-event Ca^2+^ activity across test-stages* | |  |
| Fiber photometry (n=14-17 mice/stage) | bCI denoted as green lines | |  |
|  | | |  |
| **Fig. 1h** | | |  |
|  | *Number of CS-related Ca^2+^ transients across test-stages* | |  |
| Fiber photometry (n=14-17 mice/stage) | ANOVA *F*(2.430, 49.42) = 8.918, *P* = 0.0002*  2-tailed unpaired *t*-tests: E-Ext vs L-Ext *t*(32) = 2.386, *P* = 0.0231*, E-Ext vs E-Ret *t*(32) = 2.331, *P* = 0.0262*, E-Ext vs F-Ren *t*-test: *t*(29) = 1.828, *P* = 0.0778, Ren vs L-Ext *t*-test: *t(*29) = 4.519, *P* < 0.0001*, E-Ext vs F-Ren *t*(29) = 4.342, *P* = 0.0002* | |  |
| **Fig. 1i** | | |  |
|  | *Correlation of freezing (%) and number of Ca^2+^ transients across test-stages* | |  |
| Fiber photometry (n=14-17 mice/stage) | 2-tailed Pearson’s correlation *r* = 0.9739, *P* = 0.0261* | |  |
|  | | |  |
| **Fig. 1k** | | |  |
|  | *Peri-event Ca^2+^ activity during E-Ext* | |  |
| Fiber photometry (n=5 mice) | bCI denoted as corresponding green (dark astrocytes, light neurons) lines. Benjamini-Hochberg corrected PT difference between neurons and astrocytes, denoted as orange line | |  |
|  | *CS onset latency during E-Ext* | |  |
|  | Astrocytes vs neurons, 2-tailed unpaired *t*-test: *t*(8) = 4.471, *P* = 0.0021* | |  |
|  |  | |  |
| **Fig. 1o** | | |  |
|  | *Number of CS-related Ca^2+^ transients across test-stages* | |  |
| 2-photon imaging (n=257 AQuA events/n=6 mice) | 1-way ANOVA *F*(3, 12) = 3.734, *P* = 0.0418*  2-tailed unpaired *t*-tests Pre-Con vs E-Ext *t*(8) = 3.070, *P* = 0.0154*, Pre-Con vs L-Ext *t*(8) = 0.9742, *P* = 0.3585, Pre-Con vs E-Ret *t*(8) = 0.8853, *P* = 0.4008, E-Ext vs L-Ext *t*(8) = 2.489, *P* = 0.0376*, E-Ext vs E-Ret *t*(8) = 1.283, *P* = 0.2355 | |  |
|  | | |  |
| **Fig. 1p** | | |  |
|  | *Decoding CS-presentation (F1 score)* | |  |
| 2-photon imaging (n=110 AQuA events/n=6 mice) | Observed vs shuffled data 1-tailed paired *t*-test: *t*(4)=2.719, *P* = 0.0265* | |  |
|  |  | |  |
|  | | |  |
|  | | |  |
| Related to **Fig. 2** | | | |
| **Fig. 2b** | | | |
|  | *% mCherry+ cells* | | |
| hM3Dq expressing cell counts (n=12 sections/4 mice) | %GFAP+ vs %NeuN+ cells  2-tailed paired *t*-test: *t*(11) = 25.51, *P* < 0.0001* | | |
|  | | | |
| **Fig. 2c** | | | |
|  | *Ca^2+^ transients’ cumulative frequency* | | |
| hM3Dq-actuation in neutral cage (Control n=7, hM3Dq n=9 mice) | Control vs hM3Dq 30 min after CNO: 2-tailed unpaired *t*-test *t*(14) = 3.434, *P* = 0.004* | | |
|  | | | |
| **Fig. 2e** | | | |
|  | *% Freezing* | | |
|  | *%Freezing during conditioning* | *%Freezing during extinction* | |
| Pre-extinction hM3Dq-actuation (Control n=9, hM3Dq n=11 mice) | Virus x CS 2-way ANOVA:  Virus: *F*(1, 18) = 0.001242, *P* = 0.9723  CS: *F*(2, 36) = 242.5, *P* < 0.0001*  Interaction: *F*(2, 36) = 0.3466, *P* = 0.7094  Šídák’s *post hoc* tests:  Control group CS1 vs CS3: *P* < 0.0001*  hM3Dq group CS1 vs CS3: *P* < 0.0001*  Control vs hM3Dq during CS3: *P* = 0.5770 | Virus x CS trial-block 2-way ANOVA:  Virus: *F*(1, 18) = 0.7135 *P* = 0.4094  CS trial-block: *F*(10, 180) = 33.39, *P* < 0.0001*  Interaction: *F*(10, 180) = 4.860, P < 0.0001*  Šídák’s *post hoc* tests:  Control vs hM3Dq  Block 1: *P* < 0.0001*  Block 2: *P* = 0.0067*  Blocks 3-10: *P* > 0.05 | |
|  | *%Freezing during extinction retrieval* | *%Freezing during fear renewal* | |
|  | Virus x CS 2-way ANOVA:  Virus: *F*(1, 18) = 15.16, *P* = 0.0011*  CS: *F*(1, 18) = 91.64, *P* < 0.0001*  Interaction: *F*(1, 18) = 18.10, *P* = 0.0005*  Fisher’s LSD *post hoc* test:  Control vs hM3Dq during E-Ret: *P* < 0.0001* | Virus x CS 2-way ANOVA:  Virus: *F*(1, 18) = 0.3390, *P* = 0.5676  CS: *F*(1, 18) = 103.4, *P* < 0.0001*  Interaction: *F*(1, 18) = 0.1051, *P* = 0.7495  Fisher’s LSD *post hoc* test:  Control vs hM3Dq during F-Ren: *P* = 0.7301 | |
|  | | | |
| **Fig. 2f** | | | |
|  | *Ca^2+^ events cumulative frequency during extinction training* | | |
| Pre-extinction hM3Dq-actuation (Control n=7, hM3Dq n=9 mice/session) | Control vs hM3Dq at the end of extinction training: 2-tailed unpaired *t*-test *t*(30) = 4.811, *P* < 0.0001* | | |
|  | | | |
| **Fig. 2h** | | | |
|  | *%mCherry+ cells* | | |
| hM4Di expressing cell counts (n=10 sections/3 mice) | %GFAP+ vs %NeuN+ cells  2-tailed paired *t*-test: *t*(9) = 62.12, *P* < 0.0001* | | |
|  | | | |
| **Fig. 2i** | | | |
|  | *Ca^2+^ events cumulative frequency* | | |
| hM4Di-actuation in neutral cage (Control n=8, hM4Di n=6 mice) | Control vs hM4Di 30 min after CNO: 2-tailed unpaired *t*-test *t*(11) = 2.424, *P* = 0.0338* | | |
|  | | | |
| **Fig. 2k** | | | |
|  | *% Freezing* | | |
|  | *%Freezing during conditioning* | *%Freezing during extinction* | |
| Pre-extinction hM4Di-actuation (Control n=9, hM4Di n=12 mice) | Virus x CS 2-way ANOVA:  Virus: *F*(1, 19) = 0.08998, *P* = 0.7675  CS: *F*(2, 38) = 95.06, *P*<0.0001*  Interaction: *F*(2, 38) = 0.3466, *P* = 0.7093  Šídák’s *post hoc* tests:  Control group CS1 vs CS3: *P* < 0.0001*  hM4Di group CS1 vs CS3: *P* < 0.0001*  Control vs hM4Di during CS3: *P* = 0.4591 | Virus x CS trial-block 2-way ANOVA:  Virus: *F*(1, 19) = 4.126   *P* = 0.0565  CS trial-block: *F*(10, 190) = 33.43, *P* < 0.0001*  Interaction: *F*(10, 190) = 1.335, P = 0.2144  Šídák’s *post hoc* tests:  Control vs hM4Di  Block 1: *P* = 0.0048*  Block 2: *P* = 0.0250*  Blocks 3-10: *P* > 0.05 | |
|  | *%Freezing during extinction retrieval* | *%Freezing during fear renewal* | |
|  | Virus x CS 2-way ANOVA:  Virus: *F*(1, 19) = 6.915, *P* = 0.0165*  CS: *F*(1, 19) = 74.45,*P* < 0.0001*  Interaction: *F*(1, 19) = 7.845, *P* = 0.0114*  Fisher’s LSD *post hoc* test:  Control vs hM4Di during E-Ret: *P* = 0.0005* | Virus x CS 2-way ANOVA:  Virus: *F*(1, 19) = 0.8977, *P* = 0.3553  CS: *F*(1, 18) = 103.4, *P* < 0.0001*  Interaction: *F*(1, 19) = 52.05, *P* < 0.0001*  Fisher’s LSD *post hoc* test:  Control vs hM4Di during F-Ren: *P* = 0.6662 | |
|  | | | |
| **Fig. 2l** | | | |
|  | *Ca^2+^ events cumulative frequency during extinction training* | | |
| Pre-extinction hM4Di-actuation (Control n=8, hM4Di n=6 mice/session) | Control vs hM4Di at the end of extinction training: 2-tailed unpaired *t*-test *t*(26) = 9.494, *P* < 0.0001* | | |
|  | | | |

| Related to **Fig. 3** | | |
| --- | --- | --- |
| **Fig. 3e** | | |
|  | *%Conditionally CS-responsive neurons* | |
| Conditionally CS-excited neurons (Veh n=14 neurons/8 mice, CNO n=4 neurons/7 mice) | 2-sided Chi-square test: χ^2^(1) = 5.164, *P* = 0.0231* | |
| Conditionally CS-inhibited neurons (Veh n=21 neurons/8 mice, CNO n=11 neurons/7 mice) | 2-sided Chi-square test: χ^2^(1) = 2.732, *P* = 0.1368 | |
| Stably CS-excited neurons (Veh n=9 neurons/8 mice, CNO n=9 neurons/7 mice) | 2-sided Chi-square test: χ^2^(1) = 0.0221, *P* = 0.8819 | |
| Non-modulated neurons (Veh n=138 neurons/8 mice, CNO n=146 neurons/7 mice) | 2-sided Chi-square test: χ^2^(1) = 5.705, *P* = 0.0169* | |
|  | | |
| **Fig. 3h** | | |
|  | *%CS-responsive neurons* | |
| Positively CS-modulated neurons (Veh n=42 neurons/8 mice, CNO n=20 neurons/7 mice) | 2-sided Chi-square test: χ^2^(1) = 10.06, *P* = 0.0015* | |
| CS non-modulated neurons (Veh n=267 neurons/8 mice, CNO=308 neurons/7 mice) | 2-sided Chi-square test: χ^2^(1) = 7.765, *P* = 0.0053* | |
| Negatively CS-modulated neurons (Veh n=14 neurons/8 mice, CNO n=14 neurons/7 mice) | 2-sided Chi-square test: χ^2^(1) = 0.0264, *P* = 0.8710 | |
|  | | |
| **Fig. 3i** | | |
|  | *AUC of CS-related neuronal Ca^2+^ activity* | |
| Positively CS-modulated neurons (Veh n=42 neurons/8 mice, CNO n=20 neurons/7 mice) | CNO x CS: 2-way ANOVA:  CNO: *F*(1, 48) = 0.6733, *P* = 0.4159  CS: *F*(1, 48) = 67.31, *P* < 0.0001*  Interaction: *F*(1, 48) = 0.0951, *P* = 0.7591 | Fisher’s LSD *post hoc* tests:  Veh vs CNO pre-CS: *P* = 0.6964  Veh vs CNO post-CS: *P* = 0.4193 |
| CS non-modulated neurons (Veh n=267 neurons/8 mice, CNO=308 neurons/7 mice) | CNO x CS: 2-way ANOVA:  CNO: *F*(1, 573) = 0.8449, *P* = 0.3584  CS: *F*(1, 573) = 67.31, *P* < 0.0001*  Interaction: *F*(1, 573) = 0.0951, *P* = 0.7591 | Fisher’s LSD *post hoc* tests:  Veh vs CNO pre-CS: *P* = 0.6964  Veh vs CNO post-CS: *P* = 0.4193 |
| Negatively CS-modulated neurons (Veh n=14 neurons/8 mice, CNO n=14 neurons/7 mice) | CNO: 2-way ANOVA:  CNO: *F*(1, 26) = 3.116, *P* = 0.0893  CS: *F*(1, 26) = 26.68, *P* < 0.0001*  Interaction: *F*(1, 26) = 0.9803, *P* = 0.3225 | Fisher’s LSD *post hoc* tests:  Veh vs CNO pre-CS: *P* = 0.2154  Veh vs CNO post-CS: *P* = 0.6573 |
|  |  | |
| **Fig. 3j** | | |
|  | *Variance explained by principal components* | |
| Principal components analysis (Veh n=323 neurons/8 mice; CNO n=342 neurons/7 mice) | For PC1: 2-tailed unpaired *t*-test: *t*(8) = 14.25, *P* < 0.0001* | |
|  |  | |
| **Fig. 3l** | | |
|  | *Difference between CS-related neuronal trajectories* | |
| Distance between PC1 trajectories (Veh n=323 neurons/8 mice; CNO n=342 neurons/7 mice) | Benjamini-Hochberg corrected PT difference between observed and shuffled data denoted as orange line | |
|  |  | |
| **Fig. 3m** | | |
|  | *Neuronal features decoder performance (F1 score)* | |
| Decoding group-identity  (Veh average of 100x subsampled n=5 neurons/7 mice; CNO average of 100x subsampled n=5 neurons/7 mice) | Observed vs shuffled data  Positive modulation: 2-tailed paired *t*-test: *t*(13) = 2.504, *P* = 0.0264*  Negative modulation: 2-tailed paired *t*-test: *t*(13) = 1.019, *P* = 0.3267  Non modulation: 2-tailed paired *t*-test: *t*(13) = 0.5912, *P* = 0.5645  Standard Deviation: 2-tailed paired *t*-test: *t*(13) = 0.0159, *P* = 0.9875  PC1 variation: 2-tailed paired *t*-test: *t*(13) = 0.5347, *P* = 0.6019  PC2 variation: 2-tailed paired *t*-test: *t*(13) = 0.5429, *P* = 0.5964  PC3 variation: 2-tailed paired *t*-test: *t*(13) = 0.0068, *P* = 0.9947 | |
|  |  | |
| **Fig. 3n** |  | |
|  | *Neuronal population size decoder performance (F1 score)* | |
| Decoding by population-size  (Veh n=7 mice; CNO n=7 mice) | Observed data Veh vs CNO: 2-tailed paired *t*-test: *t*(98) = 54.29, *P* < 0.0001*  Observed vs shuffled data Veh: 2-tailed paired *t*-test: *t*(98) = 70.65, *P* < 0.0001*  Observed vs shuffled data CNO: 2-tailed paired *t*-test: *t*(98) = 67.00, *P* < 0.0001* | |
|  | | |
| **Fig. 3o** | | |
|  | *Correlation of freezing and CS-presentation decoder coefficients* | |
| Pre-fear retrieval hM3Dq-actuation (Veh n=323 neurons/7 mice; CNO n=342 neurons/7 mice) | Veh: 1-tailed Pearson’s correlation *r* = 0.7030, *P* = 0.0390*  CNO: 1-tailed Pearson’s correlation *r* = 0.4098, *P* = 0.1806 | |
|  | | |
| **Fig. 3q** | | |
|  | *AUC of CS-related neuronal Ca^2+^ activity* | |
| CS-excited neurons at E-Ret after pre-extinction hM3Dq-actuation (Veh n=5 neurons/5 mice; CNO n=7 neurons/4 mice) | Veh vs CNO CS: 1-tailed unpaired *t*-test: t(10) = 2.045, *P* = 0.0340*  Veh pre vs CS: 1-tailed unpaired *t*-test: t(8) = 5.229, *P* = 0.0004*  CNO pre vs CS: 1-tailed unpaired *t*-test: t(12) = 6.732, *P* <0.0001* | |
|  | | |
| **Fig. 3r** | | |
|  | *% CS-responsive neurons* | |
| E-Ext: neurons modulated at E-Ret (CS-excited neurons at E-Ret n=12 neurons/9 mice; non-modulated neurons on E-Ret n=210 neurons/16 mice) | 2-sided Chi-square test: χ^2^(1) = 46.31, *P* < 0.0001* | |
| L-Ext: neurons modulated at E-Ret (CS-excited neurons at E-Ret n=12 neurons/9 mice; non-modulated neurons on E-Ret n=265 neurons/16 mice) | 2-sided Chi-square test: χ^2^(1) = 180.5, *P* < 0.0001* | |
|  | | |

| Related to **Fig. 4** | |
| --- | --- |
| **Fig. 4b** | |
|  | *%TdTom+ cells* |
| CalEx expressing cell counts (n=8 sections/4 mice) | %GFAP+ vs %NeuN+ cells  2-tailed paired *t*-test: *t*(7) = 43.24, *P* < 0.0001* |
|  | |
| **Fig. 4c** | |
|  | *Ca^2+^ transients’ cumulative frequency* |
| CalEx effects on Ca^2+^ activity, neutral cage (Control n=5 Control, CalEx n=8 mice) | Control vs CalEx at end of neutral cage testing: 2-tailed unpaired *t*-test *t*(11) = 3.086, *P* = 0.0104* |
|  | |
| **Fig. 4g** | |
|  | *% CS-responsive neurons* |
| BLA→PL neuronal activity, E-Ext (Control n=12 neurons/6 mice, CalEx n=16 neurons/4 mice) | 2-sided Chi-square test: χ^2^(1) = 7.479, *P* = 0.0062* |
| Non-tagged neuronal activity, E-Ext  (Control n=95 neurons/8 mice, CalEx n=67 neurons/5 mice) | 2-sided Chi-square test: χ^2^(1) = 0.0255, *P* = 0.8732 |
|  | |
| **Fig. 4h** |  |
|  | *Freezing-related unit activity during E-Ext* |
| Freezing-related unit activity, controls  (BLA→PL n=12 neurons/6 mice, BLA n=95 neurons/8 mice) | Phototagged vs non-tagged neurons, 2-tailed unpaired *t*-test *t*(105) = 2.235, *P* = 0.0275* |
| Freezing-related unit activity, CalEx  (BLA→PL n=16 neurons/4 mice, BLA n=67 neurons/5 mice) | Phototagged vs non-tagged neurons, 2-tailed unpaired *t*-test *t*(81) = 1.173, *P* = 0.2441 |
|  | |

| Related to **Extended Data Fig. S1** | | |
| --- | --- | --- |
| **Fig. S1c** | | |
|  | *AUC for CS-related Ca^2+^ activity per 5-CS trial-block of extinction* | |
| Fiber photometry (cyto-GCaMP6f) (n=17 mice) | 1-way ANOVA *F*(3.078, 49.25) = 14.46, *P* < 0.0001*  Šídák’s *post-hoc* test: E-Ext (block 1) vs L-Ext (block 10) *P* = 0.0003* | |
|  | | |
| **Fig. S1d** | | |
|  | *Peri-event Ca^2+^ activity across extinction training 5-CS trial-blocks* | |
| Fiber photometry (cyto-GCaMP6f) (n=17 mice) | bCI denoted as green lines during each trial-block of Ext 1 and Ext 2 | |
|  | | |
| **Fig. S1e** | | |
|  | *Correlation of freezing and AUC per 5-CS trial block across test-stages* | |
| Fiber photometry (cyto-GCaMP6f) (n=14-17 mice/stage) | 2-tailed Pearson’s correlation *r* = 0.89299, *P* = 0.0551 | |
|  | | |
| **Fig. S1g** | | |
|  | *Movement onset-offset induced Ca^2+^ activity* | |
| Fiber photometry (cyto-GCaMP6f) (n=133-135 onset episodes/stage, n=158-200 offset episodes/stage, 14 mice) | bCI denoted as green lines during E-Ext and L-Ext | |
|  | | |
| **Fig. S1h** | | |
|  | *%GFP+ cells* | |
| Lck-GCaMP6f expressing cell counts  (n=13 sections/6 mice) | GFAP+ vs NeuN+ cells  2-tailed paired *t*-test: *t*(12) = 51.31, *P* < 0.0001* | |
|  | | |
| **Fig. S1j** | | |
|  | *Peri-event Ca^2+^ activity during fear conditioning* | |
| Fiber photometry (lck-GCaMP6f) (n=11 mice) | bCI denoted as green lines | |
|  | | |
| **Fig. S1k** | | |
|  | *Peri-event Ca^2+^ activity across test-stages* | |
| Fiber photometry (lck-GCaMP6f) (n=10-11 mice/stage) | bCI denoted as green lines | |
|  | | |
| **Fig. S1l** | | |
|  | *Number of Ca^2+^ transients across test-stages* | |
| Fiber photometry (lck-GCaMP6f) (n=10-11 mice/stage) | 1-way ANOVA *F*(2.328, 22.50) = 11.37, *P* = 0.0002*  Šídák’s *post hoc* tests:  E-Ext vs L-Ext *P* = 0.0365*; E-Ext vs E-Ret *P* = 0.0003*; E-Ext vs F-Ren *P* = 0.9985; L-Ext vs F-Ren *P* = 0.2173; E-Ret vs F-Ren *P* = 0.0121* | |
|  | | |
|  | | |
|  | | |
| Related to **Extended Data Fig. S2** | | |
| **Fig. S2b** | | |
|  | | *% Freezing* |
| Non-US, behavior (n=4 mice) | | 1-way ANOVA *F*(1.271, 3.814) = 2.396, *P* = 0.2053  Šídák’s *post hoc* tests:  F-Con CS3 vs CS1 *P* = 0.6291; F-Con CS3 vs Test *P* = 0.9274 |
|  | | |
| **Fig. S2c** | | |
|  | | *Peri-event Ca^2+^ activity across test-stages* |
| Non-US, fiber photometry (n=4 mice) | | bCI denoted as gray lines during F-Con and Test |
|  | | |
| **Fig. S2e** | | |
|  | | *% Freezing* |
| Non-CS, behavior (n=4 mice) | | 1-way ANOVA *F*(1.263, 3.788) = 7.372, *P* = 0.0541  Šídák’s *post hoc* tests:  F-Con CS3 vs CS1 *P* = 0.2623; F-Con CS3 vs Test *P* = 0.2153 |
|  | | |
| **Fig. S2f** | | |
|  | | *Peri-event Ca^2+^ activity across test-stages* |
| Non-CS, fiber photometry (n=4 mice) | | bCI denoted as grey lines during F-Con and Test |
|  | | |
| **Fig. S2h** | | |
|  | | *% Freezing* |
| Non-extinguished, behavior (n=9 mice) | | 1-way ANOVA *F*(3.520, 28.16) = 123.7, *P* < 0.0001*  Šídák’s *post hoc* tests:  F-Con CS3 vs CS1 *P* < 0.0001*; E-Ext vs L-Ext *P* = 0.1557; E-Ext vs E-Ret *P* = 0.9998 |
|  | | |
| **Fig. S2i** | | |
|  | | *Peri-event Ca^2+^ activity across test-stages* |
| Non-extinguished, fiber photometry (n=9 mice) | | bCI denoted as green lines during E-Ext, L-Ext, and E-Ret |
|  | | |
| **Fig. S2j** | | |
|  | | *Number of Ca^2+^ transients across test-stages* |
| Non-extinguished, fiber photometry (n=9 mice) | | 1-way ANOVA *F*(1.726, 13.81) = 4.261, *P* = 0.0408*  Šídák’s *post hoc* tests:  E-Ext vs L-Ext *P* = 0.5544; E-Ext vs E-Ret *P* = 0.4833 |
|  | | |
| **Fig. S2k** | | |
|  | | *Ca^2+^ transients’ cumulative frequency across extinction training* |
| Non-extinguished, fiber photometry (Ext n=17 from Fig. 1, non-Ext n=8 mice/session) | | Extinction group vs non extinction control at the end of extinction training:  2-tailed unpaired *t*-test *t*(48) = 5.859, *P* < 0.0001* |
|  | | |
|  | | |
|  | | |

| Related to **Extended Data Fig. 3** |
| --- |
| **Fig. S3c** |

|  | *Peri-event Ca^2+^ activity during fear conditioning* |
| --- | --- |
| Fiber photometry in neurons (hSyn-GCaMP7f) (n=10 mice) | bCI denoted as green lines |

|  |
| --- |

| **Fig. S3d** | |
| --- | --- |
|  | *AUC of peri-event Ca^2+^ activity during fear conditioning* |
| Fiber photometry in neurons (hSyn-GCaMP7f) (n=10 mice) | F-Con CS vs US, 2-tailed paired *t*-test: t(9) = 6.168, *P* = 0.0002* |

|  |
| --- |

| **Fig. S3e** | |
| --- | --- |
|  | *Latency to peak CS- or shock-related Ca^2+^ activity during fear conditioning* |
| Fiber photometry in neurons (hSyn-GCaMP7f, n=10 mice) or astrocytes (cyto-GCaMP6f, n=15 mice) | Mixed effects analysis *P* < 0.0001*, Stimulus × Group interaction: *P* = 0.0017*  Fisher’s LSD post *hoc* tests:  CS: *P* < 0.0001*  Shock: *P* = 0.0876 |

|  |
| --- |

| **Fig. S3f** | |
| --- | --- |
|  | *Peri-event Ca^2+^ activity across test-stages* |
| Fiber photometry in neurons (hSyn-GCaMP7f) (n=10 mice) | bCI denoted as green lines |
|  | |
| **Fig. S3g** | |
|  | *AUC of peri-event Ca^2+^ activity across test-stages* |
| Fiber photometry in neurons (hSyn-GCaMP7f) (n=10 mice) | 1-way ANOVA *F*(2.164, 19.48) = 5.808, *P* = 0.0093*  Šídák’s *post hoc* tests:  E-Ext vs L-Ext *P* = 0.0495*; E-Ext vs E-Ret *P* = 0.0378*; E-Ext vs F-Ren *P* > 0.9999; L-Ext vs F-Ren *P* = 0.1442; E-Ret vs F-Ren *P* = 0.1948 |

|  |
| --- |

| **Fig. S3h** | |
| --- | --- |
|  | *Latency to peak CS-related Ca^2+^ activity across test-stages* |
| Fiber photometry in neurons (hSyn-GCaMP7f) (n=10 mice) or astrocytes (cyto-GCaMP6f, n=15 mice) | 2-way ANOVA:  Group: *F*(1, 23) = 138.4, *P* < 0.0001*  Stage: *F*(3, 67) = 2.926, *P* = 0.0401*  Stage × Group interaction: *F*(3, 67) = 0.7176, P = 0.5449  Šídák’s *post hoc* tests:  E-Ext *P* < 0.0001*; L-Ext *P* < 0.0001*; E-Ret *P* < 0.0001*; F-Ren *P* < 0.0001* |

|  | | | |  |
| --- | --- | --- | --- | --- |
| **Fig. S3k** | | | |  |
|  | | *Peri-event Ca^2+^ activity during fear conditioning* | |  |
| Simultaneous fiber photometry in neurons (hSyn-GCaMP6f) or astrocytes (cyto-GCaMP6f) (n=5 mice) | | bCI denoted as green (dark astrocytes, light neurons) lines during F-Con. Benjamini-Hochberg corrected PT difference between neurons and astrocytes, denoted as orange line | |  |
|  | | | |  |
| **Fig. S3l** | | | |  |
|  | | *AUC of peri-event Ca2+ activity during fear conditioning* | |  |
| Simultaneous fiber photometry in neurons (hSyn-GCaMP6f) or astrocytes (cyto-GCaMP6f) (n=5 mice) | | 2-way ANOVA:  Group: *F*(1, 8) = 3.568, *P* = 0.0956  Stimulus: *F*(1, 8) = 38.17, *P* = 0.0003*  Stimulus × Group interaction: *F*(1, 8) = 2.994, P = 0.1218  Fisher’s LSD post *hoc* tests:  CS: *P* = 0.8829  Shock: *P* = 0.0210* | |  |
|  | | | |  |
| **Fig. S3m** | | | |  |
|  | | *Latency to peak CS- or shock-related Ca^2+^ activity during fear conditioning* | |  |
| Simultaneous fiber photometry in neurons (hSyn-GCaMP6f) or astrocytes (cyto-GCaMP6f) (n=5 mice) | | 2-way ANOVA:  Group: *F*(1, 8) = 85.82, *P* < 0.0001*  Stimulus: *F*(1, 8) = 3.781, *P* = 0.0878  Stimulus × Group interaction: *F*(1, 8) = 5.565, *P* = 0.0460*  Fisher’s LSD post *hoc* tests:  CS: *P* < 0.0001*  Shock: *P* = 0.0006* | |  |
|  | | | |  |
| **Fig. S3n** | | | |  |
|  | | *AUC of peri-event Ca2+ activity during E-Ext* | |  |
| Simultaneous fiber photometry in neurons (hSyn-GCaMP6f) or astrocytes (cyto-GCaMP6f) (n=5 mice) | | 2-tailed paired *t*-test *t*(8) = 2.427, *P* = 0.0252* | |  |
|  | | | |  |
|  | | | |  |
|  | | | |  |
| Related to **Extended Data Fig. 4** | | | | |
| **Fig. S4d** | | | | |
|  | | *Ca^2+^ activity during fear conditioning* | | |
| 2-photon imaging (n=110 events/6 mice) | | bCI denoted as green lines | | |
|  | | | | |
| **Fig. S4e** | | | | |
|  | *Peri-event Ca^2+^ activity across extinction training 5-CS trial-blocks* | | | |
| 2-photon imaging (n=110 events/6 mice) | bCI denoted as green lines during each trial-block of Ext 1 and Ext 2 | | | |
|  | | | | |
| **Fig. S4f** | | | | |
|  | | *AUC for CS-related Ca^2+^ activity per 5-CS trial-blocks of extinction* | | |
| 2-photon imaging (n=110 events/6 mice) | | 1-way ANOVA *F*(2.657, 289.6) = 51.26, *P* < 0.0001*  Šídák’s *post-hoc* test: E-Ext (block 1) vs L-Ext (block 10) *P* < 0.0001* | | |
|  | | | | |
| **Fig. S4h** | | | | |
|  | | *Peri-event Ca^2+^ activity across test-stages* | | |
| 2-photon imaging (n=110 events/6 mice) | | bCI denoted as green lines during pre-Con, E-Ext, L-Ext, and E-Ret | | |
|  | | | | |
| **Fig. S4i** | | | | |
|  | | *AUC of CS-related Ca^2+^ events across test-stages* | | |
| 2-photon imaging (n=110 AQuA events/n=6 mice) | | 1-way ANOVA *F*(1.234, 134.5) = 68.31, *P* < 0.0001*  Šídák’s *post hoc* tests:  Pre-Con vs E-Ext *P* < 0.0001*; E-Ext vs L-Ext *P* < 0.0001*; E-Ext vs E-Ret *P* < 0.0001* | | |
|  | | | | |
| **Fig. S4k** | | | | |
|  | | *AUC for CS-related Ca^2+^ activity at Pre-Con → E-Ext transition* | | |
| Decreasing activity (n=16 events/3 mice) | | 2-tailed paired *t*-test: *t*(16) = 6.117, *P* < 0.0001* | | |
| Increasing activity (n=73 events/6 mice) | | 2-tailed paired *t*-test: *t*(72) = 10.85, *P* < 0.0001* | | |
| Sustained activity (n=3 events/3 mice) | | 2-tailed paired *t*-test: *t*(3) = 0.6526, *P* = 0.5810 | | |
| Non-responsive (n=2 events/2 mice) | | 2-tailed paired *t*-test: *t*(1) = 41.09, *P* = 0.0155* | | |
|  | | | | |
| **Fig. S4m** | | | | |
|  | | *AUC for CS-related Ca^2+^ activity at E-Ext → L-Ext transition* | | |
| Decreasing activity (n=48 events/5 mice) | | 2-tailed paired *t*-test: *t*(47) = 7.360, *P* < 0.0001* | | |
| Increasing activity (n=23 events/4 mice) | | 2-tailed paired *t*-test: *t*(22) = 3.184, *P* = 0.0045* | | |
| Sustained activity (n=7 events/2 mice) | | 2-tailed paired *t*-test: *t*(6) = 1.088, *P* = 0.3183 | | |
| Non-responsive (n=2 events/1 mice) | | 2-tailed paired *t*-test: *t*(1) = 1.641, *P* = 0.3484 | | |
|  | | | | |
|  | | | | |
| Related to **Extended Data Fig. 5** | | | | |
| **Fig. S5c** | | | | |
|  | | *% Freezing* | | |
|  | | *%Freezing during conditioning* | *%Freezing during extinction* | |
| CalEx effects, fear (Control n=10, CalEx n=10 mice) | | Virus x CS 2-way ANOVA:  Virus: *F*(1, 18) = 2.853, *P* = 0.1085  CS: *F*(2, 36) = 275.1, *P*<0.0001*  Interaction: *F*(2, 36) = 2.331, *P* = 0.1117  Šídák’s *post hoc* tests:  Control group CS1 vs CS3: *P* < 0.0001*  CalEx group CS1 vs CS3: *P* < 0.0001*  Control vs CalEx during CS3: *P* = 0.3250 | Virus x CS trial-block 2-way ANOVA:  Virus: *F*(1, 18) = 5.827, *P* = 0.0348*  CS trial-block: *F*(10, 180) = 48.62, *P* < 0.0001*  Interaction: *F*(10, 180) = 2.195, *P* = 0.0200  Šídák’s *post hoc* tests:  Control vs CalEx  Block 1: *P* = 0.0024*; Block 2: *P* = 0.0002*  Block 3: *P* = 0.0238*; Block 6: *P* = 0.0364*  Blocks 4,5, 7-10: *P* > 0.05 | |
|  | | *%Freezing during extinction retrieval* | *%Freezing during fear renewal* | |
|  | | Virus x CS 2-way ANOVA:  Virus: *F*(1, 18) = 5.968, *P* = 0.0251*  CS: *F*(1, 18) = 139.0, *P* < 0.0001*  Interaction: *F*(1, 18) = 15.61, *P* = 0.0009*  Fisher’s LSD *post hoc* test:  Control vs CalEx during E-Ret: *P* = 0.0001* | Virus x CS trial-block 2-way ANOVA:  Virus: *F*(1, 18) = 0.7140, *P* = 0.4092  CS trial-block: *F*(1, 18) = 98.31,*P* < 0.0001*  Interaction: *F*(1, 18) = 0.02057, *P* = 0.8875  Fisher’s LSD *post hoc* test:  Control vs CalEx during F-Ren: *P* = 0.6457 | |
|  | | | | |
| **Fig. S5d** | | | | |
|  | | *Total distance* | *%Center time* | |
| CalEx effects, novel open field (Control n=10, CalEx n=10 mice) | | 2-tailed unpaired *t*-test:  *t*(18) = 1.830, *P* = 0.0839 | 2-tailed unpaired *t*-test:  *t*(18) = 0.1177, *P* = 0.9076 | |
|  | | | | |
| **Fig. S5e** | | | | |
|  | | *Total distance* | *%Open arm time* | |
| CalEx effects, elevated plus-maze (Control n=10, CalEx n=10 mice) | | 2-tailed unpaired *t*-test:  *t*(18) = 1.452, *P* = 0.1636 | 2-tailed unpaired *t*-test:  *t*(18) = 1.385, *P* = 0.1830 | |
|  | | | | |
| **Fig. S5f** | | | | |
|  | | *Total distance* | *%Dark compartment time* | |
| CalEx effects, light-dark test (Control n=10, CalEx n=10 mice) | | 2-tailed unpaired *t*-test:  *t*(18) = 1.691, *P* = 0.1080 | 2-tailed unpaired *t*-test:  *t*(18) = 1.136, *P* = 0.2707 | |
|  | |  |  | |
| **Fig. S5h** | | | | |
|  | | *Peri-event Ca^2+^ activity during fear conditioning* | | |
| CalEx effects, fiber photometry (Control n=8, CalEx n=10 mice) | | bCI denoted as black (control) and purple (CalEx) lines during F-Con | | |
|  | | | | |
| **Fig. S5i** | | | | |
|  | | *Peri-event Ca^2+^ activity across test-stages* | | |
| CalEx effects, fiber photometry (Control n=8, CalEx n=10 mice) | | bCI denoted as black (control) and purple (CalEx) lines during E-Ext, L-Ext, E-Ret, and F-Ren. Benjamini-Hochberg corrected PT difference between control and CalEx, denoted as orange line | | |
|  | | | | |
| **Fig. S5j** | | | | |
|  | | *Number of CS-related Ca^2+^ transients across testing-stages* | | |
| CalEx effects, fiber photometry (Control n=8, CalEx n=10 mice) | | Virus x Stage 2-way ANOVA:  Virus: *F*(1, 16) = 8.170, *P* = 0.0114*  Stage: *F*(3, 48) = 4.81, *P* < 0.0001*  Interaction: *F*(3, 48) = 1.951, *P* = 0.1340  Šídák’s *post hoc* tests:  E-Ext: *P* = 0. 0008*; L-Ext: *P* = 0.4161; E-Ret: *P* = 0.0449*; F-Ren: *P* = 0.2383 | | |
|  | |  | | |
| **Fig. S5l** | | | | |
|  | | *Ca^2+^ transients’ cumulative frequency during extinction training* | | |
| CalEx effects, fiber photometry (Control n=8, CalEx n=10 mice/session) | | Control vs CalEx at the end of extinction training:  2-tailed unpaired *t*-test *t*(34) = 2.615, *P* = 0.0132* | | |
|  | | | | |

|  | | |
| --- | --- | --- |
| Related to **Extended Data Fig. S6** | | |
| **Fig. S6a** | | |
|  | *Ca^2+^ traces AUC* | |
| hM3Dq-actuation, 3 mg/kg CNO for 3 min, neutral cage (Control n=7, hM3Dq n=6 mice) | Virus x time 2-way ANOVA:  Virus: *F*(1, 11) = 6.024, *P =* 0.0320*  Time: *F*(1, 11) = 2.222, *P* = 0.1642  Interaction: *F*(1, 11) = 6.031, *P* = 0.0319*  Fisher’s LSD *post hoc* tests:  Control vs hM3Dq during pre-CNO: *P* = 0.9981  Control vs hM3Dq 10 min: *P* = 0.0022* | |
|  | | |
| **Fig. S6b** | | |
|  | *Peri-event Ca^2+^ traces* | *Ca^2+^ traces AUC* |
| hM3Dq-actuation, 3 mg/kg CNO for 3 min, CS (Control n=7, hM3Dq n=6 mice) | bCI denoted as black (control) and yellow (hM3Dq) lines. Benjamini-Hochberg corrected PT difference between control and hM3Dq, denoted as orange line | Virus x time 2-way ANOVA:  Virus: *F*(1, 11) = 0.1543, *P* = 0.7012  Time: *F*(1, 11) = 7.738, *P* = 0.0178*  Interaction: *F*(1, 11) = 0.1551, *P* = 0.7012  Fisher’s LSD *post hoc* tests:  Control vs hM3Dq pre-CS: *P* = 0.9993  Control vs hM3Dq CS: *P* = 0.5837 |
|  | | |
| **Fig. S6c** | | |
|  | *Ca^2+^ traces AUC* | |
| hM3Dq-actuation, 3 mg/kg CNO for 10 min, neutral cage (Control n=7, hM3Dq n=6 mice) | Virus x time 2-way ANOVA:  Virus: *F*(1, 11) = 14.13, *P =* 0.0032*  Time: *F*(2, 22) = 12.22, *P* = 0.0003*  Interaction: *F*(2, 22) = 15.57, *P* < 0.0001*  Šídák’s *post hoc* tests:  Control vs hM3Dq during pre-CNO: *P* = 0.9435  Control vs hM3Dq 10 min: *P* < 0.0001*  Control vs hM3Dq 20 min: *P* = 0.0306* | |
|  | | |
| **Fig. S6d** | | |
|  | *Peri-event Ca^2+^ traces* | *Ca^2+^ traces AUC* |
| hM3Dq-actuation, 3 mg/kg CNO for 3 min, CS (Control n=7, hM3Dq n=6 mice) | bCI denoted as black (control) and yellow (hM3Dq) lines. Benjamini-Hochberg corrected PT difference between control and hM3Dq, denoted as orange line | Virus x time 2-way ANOVA:  Virus: *F*(1, 11) = 4.419, *P* = 0.0594  Time: *F*(1, 11) = 14.40, *P* = 0.0030*  Interaction: *F*(1, 11) = 4.463, *P* = 0.0583  Fisher’s LSD *post hoc* tests:  Control vs hM3Dq pre-CS: *P* = 0.9944  Control vs hM3Dq CS: *P* = 0.0069* |
|  | | |
| **Fig. S6e** | | |
|  | *Ca^2+^ traces AUC* | *Number of Ca^2+^ transients* |
| hM3Dq-actuation, 3 mg/kg CNO, neutral cage (Control n=7, hM3Dq n=6 mice) | Virus x time 2-way ANOVA:  Virus: *F*(1, 11) = 39.72, *P* < 0.0001*  Time: *F*(3, 33) = 18.29, *P* < 0.0001*  Interaction: *F*(3, 33) = 38.03, *P* < 0.0001*  Šídák’s *post hoc* tests:  Control vs hM3Dq during pre-CNO: *P* = 0.9695  Control vs hM3Dq 10 min: *P* < 0.0001*  Control vs hM3Dq 20 min: *P* = 0.0013*  Control vs hM3Dq 30 min: *P* = 0.9447 | Virus x time 2-way ANOVA:  Virus: *F*(1, 11) = 0.3524, *P* = 0.5647  Time: *F*(3, 33) = 57.28, *P* < 0.0001*  Interaction: *F*(3, 33) = 20.96, *P* < 0.0001*  Šídák’s *post hoc* tests:  Control vs hM3Dq during pre-CNO: *P* = 0.338  Control vs hM3Dq 10 min: *P* = 0.0002*  Control vs hM3Dq 20 min: *P* = 0.0138*  Control vs hM3Dq 30 min: *P* = 0.0200* |
|  | | |
| **Fig. S6f** | | |
|  | *Peri-event Ca^2+^ traces* | *Ca^2+^ traces AUC* |
| hM3Dq-actuation, 3 mg/kg CNO, 0.4 mA shock (Control n=4, hM3Dq n=3 mice) | bCI denoted as black (control) and yellow (hM3Dq) lines. Benjamini-Hochberg corrected PT difference between control and hM3Dq, denoted as orange line | Virus x time 2-way ANOVA:  Virus: *F*(1, 5) = 12.23, *P* = 0.0173*  Time: *F*(1, 5) = 27.61, *P* = 0.0033*  Interaction: *F*(1, 5) = 12.15, *P* = 0.0175*    Fisher’s LSD *post hoc* test:  Control vs hM3Dq: *P* = 0.0006* |
|  | | |
| **Fig. S6g** | | |
|  | *Peri-event Ca^2+^ traces* | *Ca^2+^ traces AUC* |
| hM3Dq-actuation, 3 mg/kg CNO, 1.0 mA shock (Control n=3, hM3Dq n=3 mice) | bCI denoted as black (control) and yellow (hM3Dq) lines. Benjamini-Hochberg corrected PT difference between control and hM3Dq, denoted as orange line | Virus x time 2-way ANOVA:  Virus: *F*(1, 4) = 55.27, *P* = 0.0017*  Time: *F*(1, 4) = 64.67, *P* = 0.0013*  Interaction: *F*(1, 4) = 55.18, *P* = 0.0018*  Fisher’s LSD *post hoc* test:  Control vs hM3Dq: *P* < 0.0001* |
|  | | |
| **Fig. S6i** | | |
|  | *Ca^2+^ traces AUC* | *Number of Ca^2+^ transients* |
| hM3Dq-actuation, 0.1 mg/kg CNO, neutral cage (Control n=4, hM3Dq n=5 mice) | Virus x time 2-way ANOVA:  Virus: *F*(1, 7) = 1.049, *P* = 0.3398  Time: *F*(3, 21) = 6.799, *P* = 0.0022*  Interaction: *F*(3, 21) = 4.549, *P* = 0.0132*  Šídák’s *post hoc* tests:  Control vs hM3Dq during pre-CNO: *P* = 0.9975  Control vs hM3Dq 10 min: *P* = 0.6607  Control vs hM3Dq 20 min: *P* = 0.0026*  Control vs hM3Dq 30 min: *P* = 0.2769 | Virus x time 2-way ANOVA:  Virus: *F*(1, 7) = 10.14, *P* = 0.0154*  Time: *F*(3, 21) = 8.072, *P* = 0.0009*  Interaction: *F*(3, 21) = 3.951, *P* = 0.0222*  Šídák’s *post hoc* tests:  Control vs hM3Dq during pre-CNO: *P* = 0.5063  Control vs hM3Dq 10 min: *P* = 0.0058*  Control vs hM3Dq 20 min: *P* = 0.0003*  Control vs hM3Dq 30 min: *P* = 0.0458* |
|  | | |
| **Fig. S6j** | | |
|  | *Peri-event Ca^2+^ traces* | *Ca^2+^ traces AUC* |
| hM3Dq-actuation, 0.1 mg/kg CNO, 0.4 mA shock (Control n=4, hM3Dq n=5 mice) | bCI denoted as black (control) and light yellow (hM3Dq) lines. Benjamini-Hochberg corrected PT difference between control and hM3Dq, denoted as orange line | Virus x time 2-way ANOVA:  Virus: *F*(1, 7) = 4.012, *P* = 0.0852  Time: *F*(1, 7) = 15.25, *P* = 0.0059*  Interaction: *F*(1, 7) = 4.016, *P* = 0.0851  Fisher’s LSD *post hoc* test:  Control vs hM3Dq: *P* = 0.0133* |
|  | | |
| **Fig. S6k** | | |
|  | *Ca^2+^ traces AUC* | *Number of Ca^2+^ transients* |
| hM3Dq-actuation, 3 mg/kg CNO, diluted virus, neutral cage (Control n=4, hM3Dq 1:8 mice n=4) | Virus x time 2-way ANOVA:  Virus: *F*(1, 6) = 2.581, *P* = 0.1593  Time: *F*(3, 18) = 1.744, *P* = 0.1938  Interaction: *F*(3, 18) = 3.131, *P* = 0.0513  Šídák’s *post hoc* tests:  Control vs hM3Dq during pre-CNO: *P* > 0.9999  Control vs hM3Dq 10 min: *P =* 0.0459*  Control vs hM3Dq 20 min: *P* = 0.2697  Control vs hM3Dq 30 min: *P* = 0.9799 | Virus x time 2-way ANOVA:  Virus: *F*(1, 6) = 3.068, *P* = 0.1304  Time: *F*(3, 18) = 3.601, *P* = 0.0338*  Interaction: *F*(3, 18) = 1.073, *P* = 0.3854  Šídák’s *post hoc* tests:  Control vs hM3Dq during pre-CNO: *P* = 0.9998  Control vs hM3Dq 10 min: *P =* 0.0448*  Control vs hM3Dq 20 min: *P* = 0.2723  Control vs hM3Dq 30 min: *P* = 0.9439 |
|  | | |
| **Fig. S6l** | | |
|  | *Peri-event Ca^2+^ traces* | *Ca^2+^ traces AUC* |
| hM3Dq-actuation, 3 mg/kg CNO, diluted virus, 0.1 mA shock (Control n=4, hM3Dq 1:8 mice n=4) | bCI denoted as black (control) and yellow (hM3Dq) lines. Benjamini-Hochberg corrected PT difference between control and hM3Dq, denoted as orange line | Virus x time 2-way ANOVA:  Virus: *F*(1, 6) = 1.312, *P* = 0.2957  Time: *F*(1, 6) = 23.99, *P* = 0.0027*  Interaction: *F*(1, 6) = 1.307, *P* = 0.2965  Fisher’s LSD *post hoc* test:  Control vs hM3Dq: P = 0.1316 |
|  | | |
| **Fig. S6m** | | |
|  | *% Freezing* | |
|  | *%Freezing during conditioning* | *%Freezing during extinction* |
| Pre-extinction hM3Dq-actuation, 0.1 mg/kg CNO (Control n=12, hM3Dq n=9 mice) | Virus x CS 2-way ANOVA:  Virus: *F*(1, 19) = 1.493, *P* = 0.2510  CS: *F*(2, 38) = 157.7, *P*<0.0001*  Interaction: *F*(2, 38) = 1.434, *P* = 0.7093  Šídák’s *post hoc* tests:  Control group CS1 vs CS3: *P* < 0.0001*  hM3Dq group CS1 vs CS3: *P* < 0.0001*  Control vs hM3Dq during CS3: *P* = 0.0532 | Virus x CS trial-block 2-way ANOVA:  Virus: *F*(1, 19) = 1.953, *P* = 0.1784  CS trial-block: *F*(10, 190) = 47.10, *P* < 0.0001*  Interaction: *F*(10, 190) = 1.041, *P* = 0.4109  Šídák’s *post hoc* tests:  Control vs hM3Dq  Blocks 1,2,5-10: *P* > 0.05;  Block 3: *P* = 0.0165*; Block 4: *P* = 0.0432* |
|  | *%Freezing during extinction retrieval* | *%Freezing during fear renewal* |
|  | Virus x CS 2-way ANOVA:  Virus: *F*(1, 19) = 1.053, *P* = 0.3178  CS: *F*(1, 19) = 68.73,*P* < 0.0001*  Interaction: *F*(1, 19) = 0.9277, *P* = 0.3476  Fisher’s LSD *post hoc* test:  Control vs hM3Dq during E-Ret: *P* = 0.1675 | Virus x CS 2-way ANOVA:  Virus: *F*(1, 19) = 0.1733, *P* = 0.6818  CS: *F*(1, 19) = 20.97, *P* = 0.0002*  Interaction: *F*(1, 19) = 3.564, *P* = 0.0744  Fisher’s LSD *post hoc* test:  Control vs hM3Dq during F-Ren: *P* = 0.1202 |
|  | | |
| **Fig. S6n** | | |
|  | *Total distance* | *%Center time* |
| hM3Dq-actuation, 0.1 mg/kg CNO, novel open field (Control n=12, hM3Dq n=9 mice) | 2-tailed unpaired *t*-test:  *t*(19) = 0.6691, *P* = 0.5115 | 2-tailed unpaired *t*-test:  *t*(21) = 0.3842, *P* = 0.7051 |
|  | | |
| **Fig. S6o** | | |
|  | *% Freezing* | |
|  | *%Freezing during conditioning* | *%Freezing during extinction* |
| Pre-extinction hM3Dq-actuation, 3 mg/kg CNO, diluted virus (Control n=11, hM3Dq 1:8 n=12 mice) | Virus x CS 2-way ANOVA:  Virus: *F*(1, 21) = 0.5001, *P* = 0.4872  CS: *F*(2, 42) = 99.54, *P*<0.0001*  Interaction: *F*(2, 42) = 0.05296, *P* = 0.9485  Šídák’s *post hoc* tests:  Control group CS1 vs CS3: *P* < 0.0001*  hM3Dq group CS1 vs CS3: *P* < 0.0001*  Control vs hM3Dq during CS3: *P* = 0.5632 | Virus x CS trial-block 2-way ANOVA:  Virus: *F*(1, 21) = 0.2633, *P* = 0.6132  CS trial-block: *F*(2, 42) = 59.00, *P* < 0.0001*  Interaction: *F*(2, 42) = 0.4495, *P* = 0.6410  Šídák’s *post hoc* tests:  Control vs hM3Dq  Blocks 1-10: *P* > 0.05 |
|  | *%Freezing during extinction retrieval* | *%Freezing during fear renewal* |
|  | Virus x CS 2-way ANOVA:  Virus: *F*(1, 21) = 0.0118, *P* = 0.9147  CS: *F*(1, 21) = 78.96, *P* < 0.0001*  Interaction: *F*(1, 21) = 0.2115, *P* = 0.6503  Fisher’s LSD *post hoc* test:  Control vs hM3Dq during E-Ret: *P* = 0.7038 | Virus x CS trial-block 2-way ANOVA:  Virus: *F*(1, 21) = 0.0024, *P* = 0.9615  CS trial-block: *F*(1, 21) = 86.35,*P* < 0.0001*  Interaction: *F*(1, 21) = 0.5394, *P* = 0.4708  Fisher’s LSD *post hoc* test:  Control vs hM3Dq during F-Ren: *P* = 0.7458 |
|  | | |
| **Fig. S6p** | | |
|  | *Total distance* | *%Center time* |
| hM3Dq-actuation, 3 mg/kg CNO, diluted virus, novel open field (Control n=11, hM3Dq 1:8 n=12 mice) | 2-tailed unpaired *t*-test:  *t*(21) = 1.252, *P* = 0.2244 | 2-tailed unpaired *t*-test:  *t*(21) = 1.869, *P* = 0.0756 |
|  | | |
| Related to **Extended Data Fig. S7** | | |
| **Fig. S7b** | | |
|  | *Peri-event Ca^2+^ activity across test-stages* | |
| Pre-extinction hM3Dq-actuation (Control n=6-7, hM3Dq n=9 mice/stage) | bCI denoted as black (control) and yellow (hM3Dq) lines during E-Ext, L-Ext, E-Ret, and F-Ren. Benjamini-Hochberg corrected PT difference between control and hM3Dq, denoted as orange line | |
|  | | |
| **Fig. S7c** | | |
|  | *Number of CS-related Ca^2+^ transients across test-stages* | |
| Pre-extinction hM3Dq-actuation (Control n=6-7, hM3Dq n=9 mice/stage) | Virus x Stage 2-way ANOVA:  Virus: *F*(1, 55) = 16.83, *P* = 0.0001*  Stage: *F*(3, 55) = 31.09, *P* < 0.0001*  Interaction: *F*(3, 55) = 13.34, *P* < 0.0001*  Šídák’s *post hoc* tests:  Control vs hM3Dq during E-Ext: *P* < 0.0001*; Control vs hM3Dq during L-Ext: *P* = 0.0072*  Control vs hM3Dq during E-Ret: *P* = 0.3910; Control vs hM3Dq during F-Ren: *P* = 0.0435* | |
|  | | |
| **Fig. S7d** | | |
|  | *Freezing (%)* | *Flinching (a.u.)* |
| hM3Dq-actuation, shock-related freezing, flinching (Control n=6, hM3Dq n=6 mice) | 2-tailed unpaired *t*-test:  *t*(10) = 0.6666, *P* = 0.5210 | 2-tailed unpaired *t*-test:  *t*(10) = 1.480, *P* = 0.1696 |
|  |  | |
| **Fig. S7e** | | |
|  | *% Freezing* | |
|  | *%Freezing during conditioning* | *%Freezing during extinction* |
| Pre-fear conditioning hM3Dq-actuation (Control n=11, hM3Dq n=12 mice) | Virus x CS 2-way ANOVA:  Virus: *F*(1, 21) = 0.0013, *P* = 09721  CS: *F*(2, 42) = 177.9, *P*<0.0001*  Interaction: *F*(2, 42) = 3.453, *P* = 0.0409*  Šídák’s *post hoc* tests:  Control group CS1 vs CS3: *P* < 0.0001*  hM3Dq group CS1 vs CS3: *P* < 0.0001*  Control vs hM3Dq during CS3: *P* = 0.0748 | Virus x CS trial-block 2-way ANOVA:  Virus: *F*(1, 21) = 0.4019, *P* = 0.5330  CS trial-block: *F*(10, 210) = 37.27, *P* < 0.0001*  Interaction: *F*(10, 210) = 1.084, *P* = 0.3759  Šídák’s *post hoc* tests:  Control vs hM3Dq  Blocks 1-10: *P* > 0.05 |
|  | *%Freezing during extinction retrieval* | *%Freezing during fear renewal* |
|  | Virus x CS 2-way ANOVA:  Virus: *F*(1, 21) = 0.8497, *P* = 0.3671  CS: *F*(1, 21) = 113.2, *P* < 0.0001*  Interaction: *F*(1, 21) = 1.062, *P* = 0.3145  Fisher’s LSD *post hoc* test:  Control vs hM3Dq during E-Ret: *P* = 0.1751 | Virus x CS 2-way ANOVA:  Virus: *F*(1, 21) = 0.00095, *P* = 0.9923  CS: *F*(1, 21) = 111.0, *P* < 0.0001*  Interaction: *F*(1, 21) = 0.3128, *P* = 0.5819  Fisher’s LSD *post hoc* test:  Control vs hM3Dq during F-Ren: *P* = 0.7464 |
|  | | |
| **Fig. S7f** | | |
|  | *% Freezing* | |
|  | *%Freezing during conditioning* | *%Freezing during extinction* |
| Post-fear conditioning hM3Dq-actuation (Control n=11, hM3Dq n=13 mice) | Virus x CS 2-way ANOVA:  Virus: *F*(1, 22) = 0.6603, *P* = 0.4252  CS: *F*(2, 44) = 133.3, *P*<0.0001*  Interaction: *F*(2, 44) = 0.2409, *P* = 0.7870  Šídák’s *post hoc* tests:  Control group CS1 vs CS3: *P* < 0.0001*  hM3Dq group CS1 vs CS3: *P* < 0.0001*  Control vs hM3Dq during CS3: *P* = 0.3250 | Virus x CS trial-block 2-way ANOVA:  Virus: *F*(1, 22) = 1.644, *P* = 0.2132  CS trial-block: *F*(10, 220) = 61.15, *P* < 0.0001*  Interaction: *F*(10, 220) = 0.4974, *P* = 0.8906  Šídák’s *post hoc* tests:  Control vs hM3Dq  Blocks 1-10: *P* > 0.05 |
|  | *%Freezing during extinction retrieval* | *%Freezing during fear renewal* |
|  | Virus x CS 2-way ANOVA:  Virus: *F*(1, 22) = 0.0013, *P* = 0.9716  CS: *F*(1, 22) = 72.85, *P* < 0.0001*  Interaction: *F*(1, 22) = 0.3127, *P* = 0.5817  Fisher’s LSD *post hoc* test:  Control vs hM3Dq during E-Ret: *P* = 0.7150 | Virus x CS 2-way ANOVA:  Virus: *F*(1, 22) = 0.2004, *P* = 0.6588  CS: *F*(1, 21) = 86.35,*P* < 0.0001*  Interaction: *F*(1, 22) = 0.0441, *P* = 0.8355  Fisher’s LSD *post hoc* test:  Control vs hM3Dq during F-Ren: *P* = 0.8234 |
|  | | |
| **Fig. S7g** | | |
|  | *Total distance* | *%Center time* |
| hM3Dq-actuation, novel open field (Control n=14, hM3Dq n=17 mice) | 2-tailed unpaired *t*-test:  *t*(29) = 0.0971, *P* = 0.9233 | 2-tailed unpaired *t*-test:  *t*(29) = 1.640, *P* = 0.1118 |
|  | | |
| **Fig. S7h** | | |
|  | *Total distance* | *%Open arm time* |
| hM3Dq-actuation, elevated plus-maze (Control n=14, hM3Dq n=17 mice) | 2-tailed unpaired *t*-test:  *t*(29) = 1.331, *P* = 0.1937 | 2-tailed unpaired *t*-test:  *t*(29) = 0.9399, *P* = 0.3550 |
|  | | |
| **Fig. S7i** | | |
|  | *Total distance* | *%Dark compartment time* |
| hM3Dq-actuation, light-dark test (Control n=17, hM3Dq n=18 mice) | 2-tailed unpaired *t*-test:  *t*(33) = 0.3825, *P* = 0.7045 | 2-tailed unpaired *t*-test:  *t*(33) = 0.1250, *P* = 0.9013 |
|  | | |
| **Fig. S7j** | | |
|  | *% Freezing* | |
|  | *%Freezing during fear conditioning* | *%Freezing during extinction* |
| Pre-extinction CNO, mCherry-expressing mice (Veh n=10, CNO n=10) | CNO x CS 2-way ANOVA:  CNO: *F*(1, 18) = 0.2376, *P* = 0.6318  CS: *F*(2, 36) = 201.4, *P* < 0.0001*  Interaction: *F*(2, 36) = 0.1208, *P* = 0.8866  Šídák’s *post hoc* tests:  Veh group CS1 vs CS3: *P* < 0.0001*  CNO group CS1 vs CS3: *P* < 0.0001*  Veh vs CNO during CS3: *P* = 0.3250 | Virus x CS trial-block 2-way ANOVA:  Virus: *F*(1, 18) = 1.723, *P* = 0.2058  CS trial-block: *F*(10, 180) = 47.92, *P* < 0.0001*  Interaction: *F*(10, 180) = 0.8802, *P* = 0.5528  Šídák’s *post hoc* tests:  Control vs hM3Dq  Blocks 1,2,4-10: *P* > 0.05;  Block 3: *P* = 0.0424* |
|  | *%Freezing during extinction retrieval* | *%Freezing during fear renewal* |
|  | CNO x CS 2-way ANOVA:  CNO: *F*(1, 18) = 0.5355, *P* = 0.4737  CS: *F*(1, 18) = 70.51, *P* < 0.0001*  Interaction: *F*(1, 18) = 0.8109, *P* = 0.3798  Fisher’s LSD *post hoc* test:  Veh vs CNO during E-Ret: *P* = 0.2559 | CNO x CS 2-way ANOVA:  CNO: *F*(1, 18) = 0.0024, *P* = 0.9615  CS: *F*(1, 18) = 49.79, *P* < 0.0001*  Interaction: *F*(1, 18) = 1.654, *P* = 0.9615  Fisher’s LSD *post hoc* test:  Veh vs CNO during F-Ret: *P* = 0.4746 |
|  | | |
| **Fig. S7k** | | |
|  | *% Freezing* | |
|  | *%Freezing during conditioning* | *%Freezing during extinction* |
| Pre-extinction Veh, hM3Dq-expressing vs control virus (Control n=11, hM3Dq n=11 mice) | Virus x CS 2-way ANOVA:  Virus: *F*(1, 20) = 0.0002, *P* = 0.9659  CS: *F*(2, 40) = 376.2, *P*<0.0001*  Interaction: *F*(2, 40) = 0.2298, *P* = 0.7957  Šídák’s *post hoc* tests:  Control group CS1 vs CS3: *P* < 0.0001*  hM3Dq group CS1 vs CS3: *P* < 0.0001*  Control vs hM3Dq during CS3: *P* = 0.6454 | Virus x CS trial-block 2-way ANOVA:  Virus: *F*(1, 20) = 0.1292, *P* = 0.7230  CS trial-block: *F*(10, 200) = 56.63, *P* < 0.0001*  Interaction: *F*(10, 200) = 1.951, *P* = 0.0405*  Šídák’s *post hoc* tests:  Control vs hM3Dq  Blocks 1-10: *P* > 0.05 |
|  | *%Freezing during extinction retrieval* | *%Freezing during fear renewal* |
|  | Virus x CS 2-way ANOVA:  Virus: *F*(1, 20) = 0.8584, *P* = 0.3652  CS: *F*(1, 20) = 30.39, *P* < 0.0001*  Interaction: *F*(1, 20) = 0.6329, *P* = 0.4356  Fisher’s LSD *post hoc* test:  Control vs hM3Dq during E-Ret: *P* = 0.2309 | Virus x CS 2-way ANOVA:  Virus: *F*(1, 20) = 0.0002, *P* = 0.9888  CS: *F*(1, 20) = 54.18, *P* < 0.0001*  Interaction: *F*(1, 20) = 0.0495, *P* = 0.8262  Fisher’s LSD *post hoc* test:  Control vs hM3Dq during F-Ren: *P* = 0.8791 |
|  | | |
| **Fig. S7l** | | |
|  | *Total distance* | *%Center time* |
| Veh in hM3Dq-expressing vs control virus, novel open field (Control n=11, hM3Dq n=11 mice) | 2-tailed unpaired *t*-test:  *t*(20) = 1.680, *P* = 0.1085 | 2-tailed unpaired *t*-test:  *t*(20) = 0.7879, *P* = 0.4400 |
|  | | |
| **Fig. S7m** | | |
|  | *Total distance* | *%Open arm time* |
| Veh in hM3Dq-expressing vs control virus, elevated plus-maze (Control n=11, hM3Dq n=11 mice) | 2-tailed unpaired *t*-test:  *t*(20) = 0.2020, *P* = 0.8419 | 2-tailed unpaired *t*-test:  *t*(20) = 1.775, *P* = 0.0910 |
|  | | |
| **Fig. S7n** | | |
|  | *Total distance* | *%Dark compartment time* |
| Veh in hM3Dq-expressing vs control virus, light-dark test (Control n=11, hM3Dq n=11 mice) | 2-tailed unpaired *t*-test:  *t*(20) = 1.204, *P* = 0.2427 | 2-tailed unpaired *t*-test:  *t*(20) = 1.037, *P* = 0.9233 |
|  | | |
|  | | |
|  | | |
| Related to **Extended Data Fig. S8** | | |
| **Fig. S8b** | | |
|  | *Peri-event Ca^2+^ activity across test-stages* | |
| Pre-extinction hM4Di-actuation (Control n=8, hM4Di n=6 mice) | bCI denoted as black (control) and blue (hM4Di) lines during E-Ext, L-Ext, E-Ret, and F-Ren. Benjamini-Hochberg corrected PT difference between control and hM4Di, denoted as orange line | |
|  | | |
| **Fig. S8c** | | |
|  | *Number of CS-related Ca^2+^ transients across test-stages* | |
| Pre-extinction hM4Di-actuation (Control n=8, hM4Di n=6 mice) | Virus x Stage 2-way ANOVA:  Virus: *F*(1, 12) = 5.062, *P* = 0.0440*  Stage: *F*(1.682, 20.19) = 42.33, *P* < 0.0001*  Interaction: *F*(3, 36) = 13.83, *P* < 0.0001*  Šídák’s *post hoc* tests:  Control vs hM4Di during E-Ext: *P* = 0.0421*; Control vs hM4Di during L-Ext: *P* < 0.0001*  Control vs hM4Di during E-Ret: *P* = 0.0368*; Control vs hM4Di during F-Ren: *P* = 0.0115* | |
|  | | |
| **Fig. S8e** | | |
|  | *Ca^2+^ traces AUC* | |
| hM4Di-actuation, neutral cage (Control n=9, hM3Dq n=5, hM4Di n=5 mice) | Virus x time 2-way ANOVA:  Virus: *F*(2, 16) = 25.56, *P < 0.0001**  Time: *F*(4, 64) = 54.00, *P < 0.0001**  Interaction: *F*(8, 64) = 23.88, *P* < 0.0001*  Šídák’s *post hoc* tests:  Control vs hM3Dq: pre-CNO *P* > 0.9999; 30 min *P* < 0.0001*; 60 min *P* < 0.0001*; 90 min *P* < 0.0001*; 120 min *P* < 0.0001*  Control vs hM4Di: pre-CNO *P* > 0.9999; 30 min *P* < 0.0001^#^; 60 min *P* = 0.0005^#^; 90 min *P* = 0.0093^#^; 120 min *P* < 0.0001^#^ | |
|  | | |
| **Fig. S8f** | | |
|  | *Ca^2+^ transients’ cumulative frequency* | |
| hM4Di-actuation, neutral cage (Control n=9, hM3Dq n=5, hM4Di n=5 mice) | Control vs hM3Dq 2 hours after CNO: 2-tailed unpaired *t*-test *t*(12) = 3.817, *P* = 0.0025*  Control vs hM4Di 2 hours after CNO: 2-tailed unpaired *t*-test *t*(12) = 5.827, *P* < 0.0001^#^ | |
|  | | |
| **Fig. S8g** | | |
|  | *% Freezing* | |
|  | *%Freezing during conditioning* | *%Freezing during extinction* |
| Pre-extinction hM3Dq-actuation (Control n=4, hM3Dq n=8 mice) | Virus x CS 2-way ANOVA:  Virus: *F*(1, 10) = 0.2189, *P* = 0.6499  CS: *F*(2, 20) = 73.429, *P*<0.0001*  Interaction: *F*(2, 20) = 0.4479, *P* = 0.6453  Šídák’s *post hoc* tests:  Control group CS1 vs CS3: *P* < 0.0001*  hM3Dq group CS1 vs CS3: *P* < 0.0001*  Control vs hM3Dq during CS3: *P* = 0.3832 | Virus x CS trial-block 2-way ANOVA:  Virus: *F*(1, 10) = 0. 9299, *P* = 0.3576  CS trial-block: *F*(10, 100) = 40.14, *P* < 0.0001*  Interaction: *F*(10, 100) = 1.224, *P* = 0.2851  Šídák’s *post hoc* tests:  Control vs hM3Dq  Blocks 1-10: *P* > 0.05 |
|  | *%Freezing during extinction retrieval*  *with CNO* | *%Freezing during extinction retrieval*  *without CNO* |
|  | Virus x CS 2-way ANOVA:  Virus: *F*(1, 10) = 0.3483, *P* = 0.5682  CS: *F*(1, 10) = 21.17, *P* = 0.0010*  Interaction: *F*(1, 10) = 0.2443, *P* = 0.6318  Fisher’s LSD *post hoc* test:  Control vs hM3Dq during E-Ret: *P* = 0.4526 | Virus x CS 2-way ANOVA:  Virus: *F*(1, 10) = 1.659, *P* = 0.2268  CS: *F*(1, 10) = 47.47, *P* < 0.0001*  Interaction: *F*(1, 10) = 0.8968, *P* = 0.3660  Fisher’s LSD *post hoc* test:  Control vs hM3Dq during E-Ret: *P* = 0.1287 |
|  | | |
| **Fig. S8h** | | |
|  | *% Freezing* | |
|  | *%Freezing during conditioning* | *%Freezing during extinction* |
| Pre-extinction hM4Di-actuation (Control n=4, hM3Di n=8 mice) | Virus x CS 2-way ANOVA:  Virus: *F*(1, 10) = 1.142, *P* = 0.3103  CS: *F*(2, 20) = 402.5, *P*<0.0001*  Interaction: *F*(2, 20) = 4.642, *P* = 0.0.021*  Šídák’s *post hoc* tests:  Control group CS1 vs CS3: *P* < 0.0001*  hM4Di group CS1 vs CS3: *P* < 0.0001*  Control vs hM4Di during CS3: *P* = 0.3359 | Virus x CS trial-block 2-way ANOVA:  Virus: *F*(1, 10) = 0.2228, *P* = 0.6471  CS trial-block: *F*(10, 100) = 31.92, *P* < 0.0001*  Interaction: *F*(10, 100) = 0.5245, *P* = 0.8693  Šídák’s *post hoc* tests:  Control vs hM3Di  Blocks 1-10: *P* > 0.05 |
|  | *%Freezing during extinction retrieval*  *with CNO* | *%Freezing during extinction retrieval*  *without CNO* |
|  | Virus x CS 2-way ANOVA:  Virus: *F*(1, 10) = 0.4910, *P* = 0.4995  CS: *F*(1, 10) = 20.02, *P* = 0.0012*  Interaction: *F*(1, 10) = 0.2439, *P* = 0.6321  Fisher’s LSD *post hoc* test:  Control vs hM4Di during E-Ret: *P* = 0.4053 | Virus x CS 2-way ANOVA:  Virus: *F*(1, 10) = 1.005, *P* = 0.3398  CS: *F*(1, 10) = 52.56, *P* < 0.0001*  Interaction: *F*(1, 10) = 1.374, *P* = 0.32682  Fisher’s LSD *post hoc* test:  Control vs hM4Di during E-Ret: *P* = 0.1396 |
|  | | |
| **Fig. S8i** | | |
|  | *Total distance* | *%Center time* |
| hM4Di-actuation, novel open field (Control n=9, hM4Di n=13 mice) | 2-tailed unpaired *t*-test:  *t(*19) = 0.3122, *P* = 0.7583 | 2-tailed unpaired *t*-test:  *t*(19) = 1.240, *P* = 0.2299 |
|  | | |
| **Fig. S8j** | | |
|  | *Total distance* | *%Open arm time* |
| hM4Di-actuation, elevated plus-maze (Control n=9, hM4Di n=13 mice) | 2-tailed unpaired *t*-test:  *t*(19) = 1.029, *P* = 0.3164 | 2-tailed unpaired *t*-test:  *t*(19) = 1.870, *P* = 0.0769 |
|  | | |
| **Fig. S8k** | | |
|  | *Total distance* | *%Dark compartment time* |
| hM4Di-actuation, light-dark test (Control n=9, hM4Di n=13 mice) | 2 tailed unpaired *t*-test:  *t*(20) = 0.06578, *P* = 0.9482 | 2-tailed unpaired *t*-test:  *t*(20) = 0.08873, *P* = 0.9302 |
|  | | |
|  | | |

| Related to **Extended Data Fig. 10** |
| --- |

| **Fig. S10b** | |
| --- | --- |
|  | *%GFP+ cells* |
| TeNT expressing cell counts (n=11 sections/3 mice) | %GFAP+ vs %NeuN+ cells  2-tailed paired *t*-test: *t*(10) = 24.11, *P* < 0.0001* |

|  | | |
| --- | --- | --- |
| **Fig. S10d** | | |
|  | *% Freezing* | |
|  | *%Freezing during conditioning* | *%Freezing during extinction* |
| TeNT effects, fear (Control n=12, TeNT n=10 mice) | Gene x CS 2-way ANOVA:  Gene: *F*(1, 20) = 0.3116, *P* = 0.5829  CS: *F*(2, 40) = 205.0, *P*<0.0001*  Interaction: *F*(2, 40) = 1.847, *P* = 0.1710  Šídák’s *post hoc* tests:  Control group CS1 vs CS3: *P* < 0.0001*  TeNT group CS1 vs CS3: *P* < 0.0001*  Control vs TeNT during CS3: *P* = 0.0644 | Virus x CS trial-block 2-way ANOVA:  Virus: *F*(1, 20) = 0.9911, *P* = 0.3314  CS trial-block: *F*(10, 200) = 20.04, *P* < 0.0001*  Interaction: *F*(10, 200) = 3.148, *P* = 0.0009*  Šídák’s *post hoc* tests:  Control vs hM3Dq  Block 1: *P* = 0.0025*;  Block 2: *P* = 0.0092*;  Blocks 3-10: *P* > 0.05 |
|  | *%Freezing during extinction retrieval* | *%Freezing during fear renewal* |
|  | Gene x CS 2-way ANOVA:  Gene: *F*(1, 20) = 13.15, *P* = 0.0017*  CS: *F*(1, 20) = 120.3, *P* < 0.0001*  Interaction: *F*(1, 20) = 8.753, *P* = 0.0078*  Fisher’s LSD *post hoc* test:  Control vs TeNT during E-Ret: *P* < 0.0001* | Gene x CS trial-block 2-way ANOVA:  Gene: *F*(1, 20) = 3.883, *P* = 0.0628  CS trial-block: *F*(1, 20) = 106.7, *P* < 0.0001*  Interaction: *F*(1, 20) = 0.0006, *P* = 0.9804  Fisher’s LSD *post hoc* test:  Control vs TeNT during F-Ren: *P* = 0.0768 |
|  | | |
| **Fig. S10e** | | |
|  | *Total distance* | *%Center time* |
| TeNT effects, novel open field (Control n=12, TeNT n=10 mice) | 2-tailed unpaired *t*-test:  *t*(20) = 1.074, *P* = 0.2956 | 2-tailed unpaired *t*-test:  *t*(20) = 0.9496, *P* = 0.3537 |
|  | | |
| **Fig. S10f** | | |
|  | *Total distance* | *%Open arm time* |
| TeNT effects, elevated plus-maze (Control n=12, TeNT n=10 mice) | 2-tailed unpaired *t*-test:  *t*(20) = 1.124, *P* = 0.2743 | 2-tailed unpaired *t*-test:  *t*(20) = 1.054, *P* = 0.3044 |
|  | | |
| **Fig. S10g** | | |
|  | *Total distance* | *%Dark compartment time* |
| TeNT effects, light-dark test (Control n=12, TeNT n=10 mice) | 2-tailed unpaired *t*-test:  *t*(20) = 0.3301, *P* = 0.7448 | 2-tailed unpaired *t*-test:  *t*(20) = 1.950, *P* = 0.0653 |
|  | | |

| Related to **Extended Data Fig. S11** | | |
| --- | --- | --- |
| **Fig. S11c** | | |
|  | *sEPSC amplitude* | |
| Neuronal *in vitro* slice recordings (n=6-9 cells/7 mice expressing hM3Dq) | Repeated measures one-way ANOVA *F*(2, 21) = 0.2933, *P* = 0.7488  Šídák’s *post hoc* tests:  Pre-CNO vs CNO 10 min *P =* 0.7234; Pre-CNO vs CNO 30 min *P =* 0.7234 | |
|  | | |
|  | | |
| **Fig. S11d** | | |
|  | *sEPSC frequency* | |
| Neuronal *in vitro* slice recordings (n=6-9 cells/7 mice expressing hM3Dq) | Repeated measures one-way ANOVA *F*(2, 21) = 1.006, *P* = 0.3826  Šídák’s *post hoc* tests:  Pre-CNO vs CNO 10 min *P =* 0.8480; Pre-CNO vs CNO 30 min *P =* 0.9468 | |
|  |  | |
| **Fig. S11e** | | |
|  | *sEPSC decay* | |
| Neuronal *in vitro* slice recordings (n=6-9 cells/7 mice expressing hM3Dq) | Repeated measures one-way ANOVA *F*(2, 21) = 0.7699, *P* = 0.4757  Šídák’s *post hoc* tests:  Pre-CNO vs CNO 10 min *P =* 0.4337; Pre-CNO vs CNO 30 min *P =* 0.9959 | |
|  | | |
| **Fig. S11g** | | |
|  | *%GFP+ cells* | |
| hSyn-GCaMP7f expressing cell counts (n=7 sections/3 mice) | %GFAP+ vs %NeuN+ cells  2-tailed paired *t*-test: *t*(6) = 45.11, *P* < 0.0001* | |
|  |  | |
| **Fig. S11h** | | |
|  | *Ca^2+^ traces AUC* | *Ca^2+^ transients’ cumulative frequency* |
| hSyn-GCaMP7f fiber photometry, neutral cage (n=11 Control, n=13 hM3Dq mice) | Virus x time 2-way ANOVA:  Virus: *F*(1, 22) = 0.6282, *P* = 0.4365  Time: *F*(3, 66) = 1.325, *P* =0.2739  Interaction: *F*(3, 66) = 1.019, *P* = 0.3898  Šídák’s *post hoc* tests:  Control vs hM3Dq during pre-CNO: *P* = 0.9969  Control vs hM3Dq 10 min: *P* = 0.9594  Control vs hM3Dq 20 min: *P* = 0.5590  Control vs hM3Dq 30 min: *P* = 0.0930 | Control vs hM3Dq 30 min after CNO:  2-tailed unpaired *t*-test  *t*(2) = 0.2700, P = 0.7879 |
|  | | |
| **Fig. S11j** | | |
|  | *Peri-event Ca^2+^ activity across test-stages* | |
| Pre-extinction hM3Dq-actuation, hSyn-GCaMP7f fiber photometry (n=11 Control, n=13 hM3Dq mice) | bCI denoted as corresponding trace color lines in the figure during E-Ext, L-Ext, E-Ret, and F-Ren. | |
|  | | |
| **Fig. S11k** | | |
|  | *AUC of CS-related Ca^2+^ transients across test-stages* | |
| Pre-extinction hM3Dq-actuation, hSyn-GCaMP7f fiber photometry (n=11 Control, n=13 hM3Dq mice) | Virus x time 2-way ANOVA:  Virus: *F*(1, 22) = 0.2681, *P* = 0.6098  Time: *F*(3, 66) = 17.29, *P* < 0.0001*  Interaction: *F*(3, 66) = 0.1356, *P* = 0.9385  Šídák’s *post hoc* tests:  Control vs hM3Dq during E-Ext: *P* = 0.8092; Control vs hM3Dq during L-Ext: *P* = 0.5242  Control vs hM3Dq during E-Ret: *P* = 0.5464; Control vs hM3Dq during F-Ren: *P* = 0.9530 | |
|  | | |

| Related to **Extended Data Fig. S12** | | | |
| --- | --- | --- | --- |
| **Fig. S12b** | | | |
|  | *% Freezing* | | |
|  | *%Freezing during pre-conditioning* | *%Freezing during conditioning* | |
| Pre-fear retrieval hM3Dq-actuation (Veh n=8, CNO n=7 mice) | Virus x CS 2-way ANOVA:  Virus: *F*(1, 13) = 0.8060, *P* = 0.3856  CS: *F*(1, 13) = 4191, *P* < 0.0001*  Interaction: *F*(1, 13) = 0.6719, *P* = 0.4272  Fisher’s LSD *post hoc* test:  Veh vs CNO during Pre-Con: *P* = 0.2436 | Virus x CS 2-way ANOVA:  Virus: *F*(1, 13) = 1.268, *P* = 0.2805  CS: *F*(2, 26) = 115.5, *P*<0.0001*  Interaction: *F*(2, 26) = 0.0209, *P* = 0.9723  Šídák’s *post hoc* tests:  Veh group CS1 vs CS3: *P* < 0.0001*  CNO group CS1 vs CS3: *P* < 0.0001*  Veh vs CNO during CS3: *P* = 0.4899 | |
|  | *%Freezing during fear retrieval*  Virus x CS 2-way ANOVA:  Virus: *F*(1, 13) = 13.97, *P* = 0.0025*  CS: *F*(1, 13) = 189.8, *P* < 0.0001*  Interaction: *F*(1, 13) = 22.48, P = 0.0004*  Fisher’s LSD *post hoc* test:  Veh vs CNO during F-Ret: *P* < 0.0001* |  | |
|  | | | |
| **Fig. S12c** | | | |
|  | *Average distance from centroid of CS-responsive neurons* | | |
| Topography of CS-responsive neurons  (Veh n=8 mice; CNO n=5 mice) | Veh vs CNO, 2-tailed unpaired *t*-test: *t*(11) = 0.3916, *P* = 0.7028 | | |
|  | | | |
| **Fig. S12d** | | | |
|  | *% Freezing* | | |
|  | *%Freezing during fear conditioning* | *%Freezing during fear retrieval* | |
| Pre-fear retrieval hM3Dq-actuation (Veh n=10, CNO n=10 mCherry-expressing mice) | CNO x CS 2-way ANOVA:  CNO: *F*(1, 18) = 0.2376, *P* = 0.6318  CS: *F*(2, 36) = 201.4, *P* < 0.0001*  Interaction: *F*(2, 36) = 0.1208, *P* = 0.8866  Šídák’s *post hoc* tests:  Veh group CS1 vs CS3: *P* < 0.0001*  CNO group CS1 vs CS3: *P* < 0.0001*  Veh vs CNO during CS3: *P* = 0.3250 | CNO x CS 2-way ANOVA:  CNO: *F*(1, 18) = 0.0157, *P* = 0.9017  CS: *F*(1, 18) = 102.8, *P* < 0.0001*  Interaction: *F*(1, 18) = 0.0047, *P* = 0.9461  Fisher’s LSD *post hoc* test:  Veh vs CNO during F-Ret: *P* = 0.8917 | |
|  | | | |
| **Fig. S12f** |  | |  |
|  | *% Freezing* | |  |
|  | *%Freezing during conditioning* | *%Freezing during extinction* |  |
| Pre-extinction hM3Dq-actuation (Veh n=6, CNO n=8 mice) | Virus x CS 2-way ANOVA:  Virus: *F*(1, 12) = 0.1228, *P* = 0.7321  CS: *F*(2, 24) = 142.3, *P* < 0.0001*  Interaction: *F*(2, 24) = 0.6655, *P* = 0.5232  Šídák’s *post hoc* tests:  Veh group CS1 vs CS3: *P* < 0.0001*  CNO group CS1 vs CS3: *P* < 0.0001*  Veh vs CNO during CS3: *P* = 0.5329 | Virus x CS trial-block 2-way ANOVA:  Virus: *F*(1, 12) = 1.528, *P* = 0.6471  CS trial-block: *F*(10, 120) = 9.262, *P* < 0.0001*  Interaction: *F*(10, 120) = 3.113, *P* = 0.0015  Šídák’s *post hoc* tests:  Control vs hM3Dq  Block 1: *P* = 0.0008*;  Block 2: *P* = 0.0197*;  Block 3: *P* = 0.0253*;  Blocks 4-10: *P* > 0.05 |  |
|  | *%Freezing during extinction retrieval* | *%Freezing during fear renewal* |  |
|  | Virus x CS 2-way ANOVA:  Virus: *F*(1, 12) = 2.996, *P* = 0.1091  CS: *F*(1, 12) = 170.3, *P* < 0.0001*  Interaction: *F*(1, 12) = 10.15, *P* = 0.0071*  Fisher’s LSD *post hoc* test:  Veh vs CNO during E-Ret: *P =* 0.0032* | Virus x CS 2-way ANOVA:  Virus: *F*(1, 12) = 1.625, *P* = 0.2666  CS: *F*(1, 12) = 138.8, *P* < 0.0001*  Interaction: *F*(1, 12) = 0.6367, *P* = 0.4404  Fisher’s LSD *post hoc* test:  Veh vs CNO during F-Ren: *P* = 0.5559 |  |
|  | | | |
| **Fig. 12g** | | | |
|  | *AUC of CS-related neuronal Ca^2+^ activity* | | |
| CS-excited neurons at E-Ret after pre-extinction hM3Dq-actuation (Veh n=5 neurons/5 mice; CNO n=7 neurons/4 mice) | Veh vs CNO CS: 2-tailed unpaired *t*-test: t(10) = 1.154, *P* = 0.2753  Veh pre vs CS: 2-tailed unpaired *t*-test: t(10) = 3.148, *P* = 0.0136*  CNO pre vs CS: 2-tailed unpaired *t*-test: t(12) = 1.719, *P* = 0.1113 | | |
|  | | | |
| **Fig. 12j** | | | |
|  | *% CS-responsive neurons* | | |
| BLA→PL neuronal activity, L-Ext (Control n=12 neurons/4 mice, CalEx n=14 neurons/2 mice) | 2-sided Chi-square test: χ^2^(1) = 0.8512, *P* = 0.3562 | | |
| Non-tagged neuronal activity, L-Ext  (Control n=82 neurons/8 mice, CalEx n=48 neurons/5 mice) | 2-sided Chi-square test: χ^2^(1) = 5.384, *P* = 0.0203* | | |
| BLA→PL neuronal activity, E-Ret (Control n=7 neurons/4 mice, CalEx n=18 neurons/3 mice) | 2-sided Chi-square test: χ^2^(1) = 2.138, *P* = 0.1437 | | |
| Non-tagged neuronal activity, E-Ret  (Control n=85 neurons/7 mice, CalEx n=46 neurons/5 mice) | 2-sidedChi-square test: χ^2^(1) = 0.6165, *P* = 0.4323 | | |
|  | | | |
|  | | | |
